# Study on partial discharge characteristics of C_6_F_12_O mixed gas

**DOI:** 10.1038/s41598-022-05427-0

**Published:** 2022-04-15

**Authors:** Xiajin Rao, Dajian Li, Xiaoxing Zhang, Xiaofei Xia, Yi Su, Yufeng Lu, Boya Peng

**Affiliations:** Electric Power Research Institute of Guangxi Power Grid Co., Ltd, Nanning, 530023 China

**Keywords:** Engineering, Materials science

## Abstract

The greenhouse effect of SF_6_ increasingly limits its application in various gas insulated equipment. C_6_F_12_O combines the advantages of insulation resistance, safety and environmental protection. When mixed with buffer gas, C_6_F_12_O is considered to have potential application prospects in medium and low voltage equipment. In this paper, about the partial discharge characteristics of the mixed gas, an experimental study was carried out. The partial discharge initiation voltage and discharge extinction voltage of mixed gas under power frequency voltage are measured and compared with the breakdown voltage. The results show that the breakdown voltage is greatly improved after adding C_6_F_12_O, with the increase of mixing ratio, the partial discharge initiation voltage and extinction voltage of mixed gas gradually increase, and the effect of gas pressure on high mixing ratio is obvious. The difference between the partial discharge inception voltage and the breakdown voltage is larger than that of pure N_2_. The research in this paper can provide an important reference for the application, operation and protection of C_6_F_12_O mixed gas in medium and low voltage equipment.

## Introduction

Gas insulated equipment is widely used in power systems due to its miniaturization and stable insulation performance. Its main insulating medium is SF_6_, which has a high Global Warming Potential (GWP). The GWP value of SF_6_ is approximately 23,500 times that of CO_2_^1^, limiting its use is the current environmental demand. Using new environmental protection gas as insulation medium has become a hot spot in the current research of the global power industry^2^.

**Figure 1:**
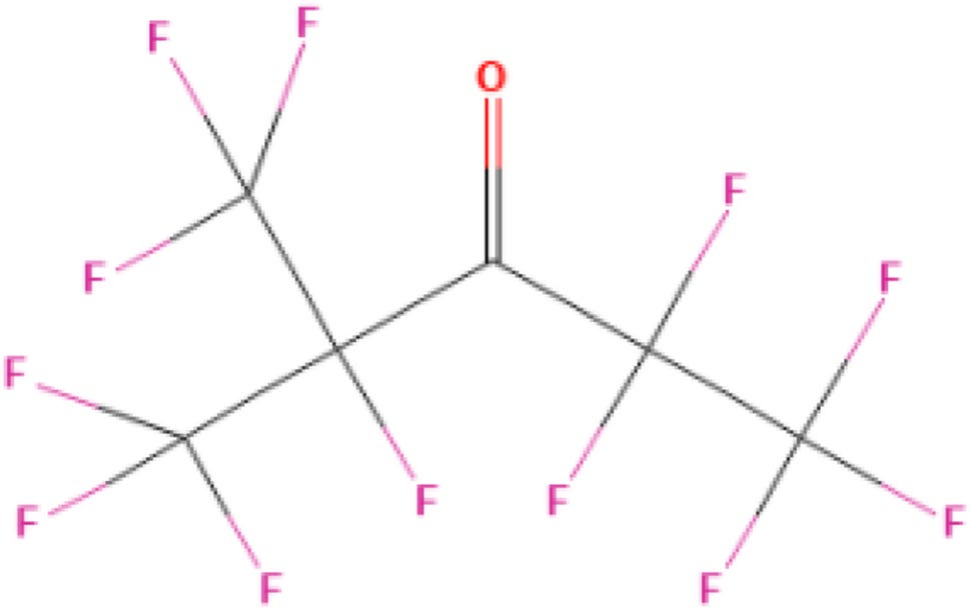
Molecular formula of C_6_F_12_O.

C_6_F_12_O of ketones fluoride is also a substance with excellent insulating properties. This substance is non-flammable, non-explosive and non-toxic. It has a boiling point of 49 ℃ under standard conditions and a molecular weight of 316. It is used as a cover gas for fire extinguishing agents, magnesium treatment, and two-phase immersion cooling. The GWP value of the substance is 1, which has no destructive effect on the ozone layer, and the dielectric strength is 1.7 times higher than SF_6_^3^. The GIS electrical performance test of 145 kV shows that adding C_6_F_12_O to the air can achieve the same insulation strength as SF_6_^4^. The molecular formula of this substance is shown in Fig. 1.

The basic parameters of C_6_F_12_O are shown in Table 1. It is worth noting that the liquefaction temperature of C_6_F_12_O is much higher than SF_6_, which is also the main reason for limiting its scope of application to medium and low pressure equipment. Therefore, it is necessary to determine the pressure value and mixing ratio that can be used in the equipment according to its saturated vapor pressure parameters.

**Table 1:** Basic parameters of C_6_F_12_O.

Molecular formula	C_6_F_12_O	SF_6_
Molecular weight	316.04	146.05
Boiling point (0.1 MPa)	49.2 °C	− 63.9
Critical parameter: critical temperature (℃)	168.7	45.55
Critical concentration (kg/m^3^)	639.1	730
Critical pressure (MPa)	1.865	3.78
Density (1 atm) kg/m^3^	13.6	6.139
Atmospheric lifetime	5 days	3200 years
ODP	0	0
GWP	Less than 1	23,900

Mantilla et al. in Switzerland found that when the mixture of C_5_F_10_O, C_6_F_12_O and air was proportional to a certain proportion, the power frequency AC breakdown voltage of the mixture can reach three times that of air and carbon dioxide. Although these are not as high as the breakdown voltage of SF_6_ under the same conditions, it can reach the level of SF_6_ at a lower pressure by increasing the pressure of the mixture. At the same time, he also observed that the mixed gas obtained by adding a small amount of C_6_F_12_O to the air has significantly improved the withstand voltage level of the air under the lightning impulse voltage^5^. Zhao et al. studied the decomposition products of C_6_F_12_O/N_2_ gas mixture and C_6_F_12_O/air mixture after corona discharge, and concluded that C_6_F_12_O/N_2_ mixture gas decomposes more products after corona discharge and may produce CF_3_CN toxic substances^6^. It is found that the breakdown voltage of 3% C_6_F_12_O/N_2_ mixture gas at atmospheric pressure is 1.7 times higher than that of pure N_2_ breakdown voltage, which is equivalent to the breakdown voltage of 10% SF_6_/N_2_ mixture gas, and there is no decreasing trend of breakdown voltage after 100 times breakdown experiments. CF_4_, C_2_F_6_, C_3_F_6_ and other fluorocarbons were obtained by analyzing the decomposition products of the mixture gas after breakdown^7^. The liquefaction temperature of C_6_F_12_O is high, which needs to be mixed with buffer gas when used as insulating material. Therefore, it can be considered to mix the substance with a single conventional gas to improve the insulation characteristics of the conventional gas.^8–10^Due to the stable chemical nature of N_2_, we need to further study the insulating properties of C_6_F_12_O/N_2_ gas mixture, and give full play to its greater application potential of the mixed gas in low-voltage equipment such as gas insulated switchgear and ring network cabinets.

Gas insulation equipment often has various kinds of insulation defects such as impurity residue and metal protrusions, which makes the local electric field become non-uniform and cause partial discharge^11^. In this paper, the partial discharge of C_6_F_12_O mixed gas at power frequency is studied. Simultaneously measure the breakdown voltage value of the mixed gas under different conditions, and compare with the partial discharge voltage to summarize the partial discharge characteristics of the mixed gas.

## Experiment

### Experimental platform

**Figure 2:**
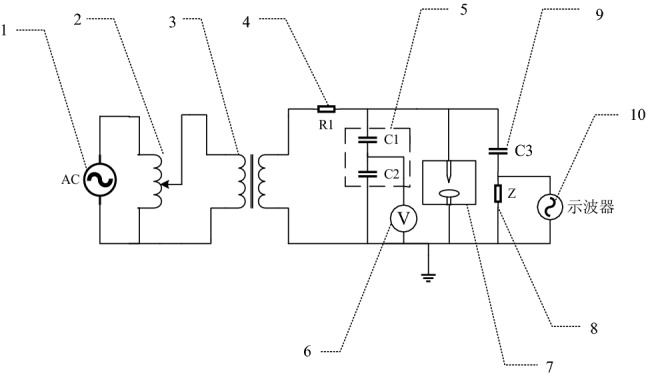
Experimental circuit diagram. 1—AC power supply 2—AC voltage regulator 3—No halo test transformer 4—Protection resistance 5—Capacitor divider 6—Voltage meter 7—Discharge chamber 8—No sense detection impedance 9—Coupling capacitor 10—Oscilloscope.

Figure 2 shows the circuit diagram of the power frequency AC experiment.

The main equipment is introduced as follows:

- *Induction voltage regulator* model TEDGC-25, working voltage 380 V, output voltage range 0–400 V, rated power 50 kVA.
- *Corona free experimental transformer* model YDTW-50VA/100 kV, connected with inductive voltage regulator, transformation ratio of 1:250 and output voltage of 0–100 kV.
- *Resistor* R = 10 k Ω, which plays a protective role in the circuit.
- *Capacitive voltage divider* C_1_/C_2_ = 2 nF/1 µ F, which converts the experimental voltage with high amplitude into the value within the range that can be directly measured by the voltmeter.
- *Discharge chamber* install electrodes and fill the experimental gas, and make the experimental gas partial discharge or breakdown under different electrodes by applying voltage, so as to detect the gas insulation performance.
- *Coupling capacitance and non inductive detection impedance* partial discharge signal is measured by pulse current method.
- *Digital storage oscilloscope* Tektronix DPO7104, with a bandwidth of 1 GHz, 4 acquisition channels and a sampling rate of 20 GS/s.

**Figure 3:**
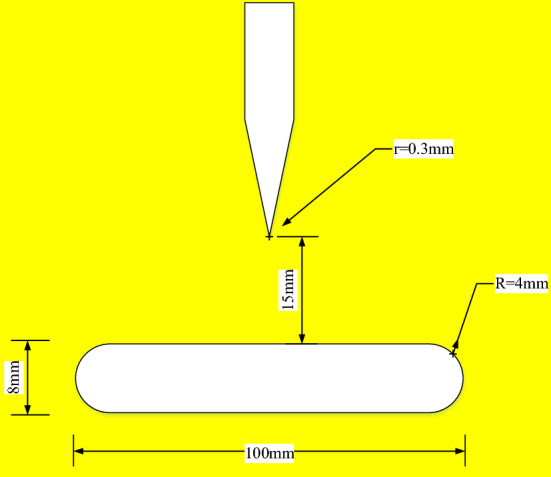
Size of the needle-plate electrode.

This experiment uses a needle plate electrode to simulate metal protrusion defects in the device. The needle plate electrode is made of brass material, the tip of needle electrode is a sphere with a radius of 0.3 mm, the radius of the plate electrode is 50 mm and the thickness is 8 mm. The gap distance between the needle tip and the upper surface of the plate electrode is set to be 15 mm constant and fixed to the closed air chamber. The electrode is shown in Fig. 3.

### Experimental process

**Figure 4:**
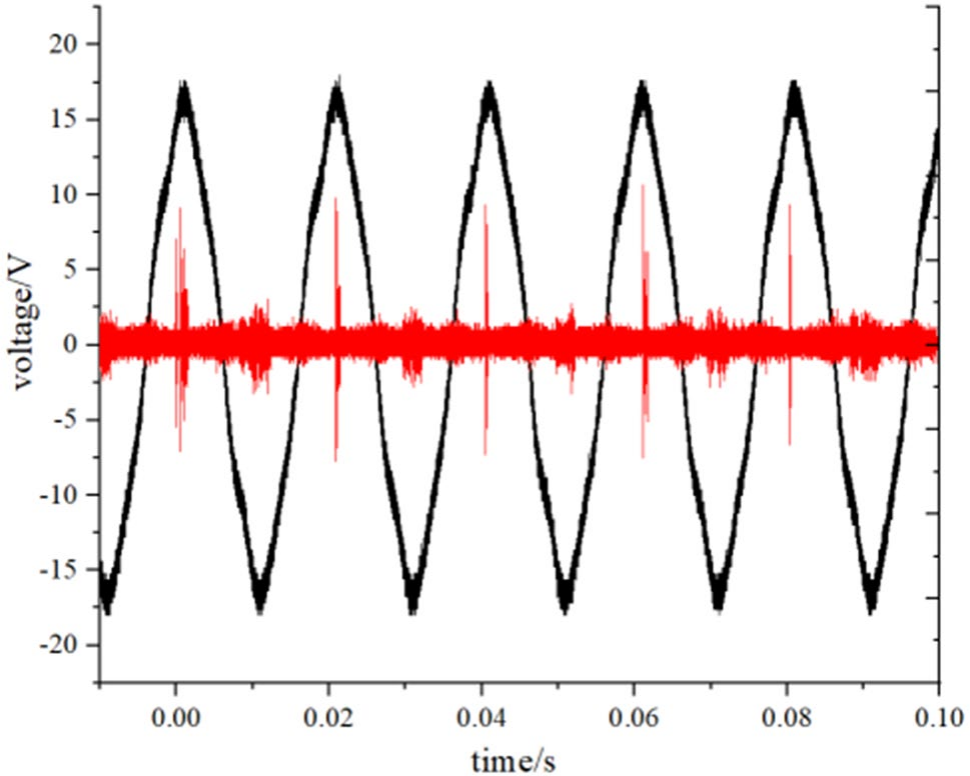
Typical partial discharge waveform.

The main measurement data of this experiment include partial discharge inception voltage, partial discharge extinction voltage^11^ and breakdown voltage. Partial discharge inception voltage (PDIV) refers to the voltage value when the partial discharge phenomenon occurs when the voltage gradually increases. Partial discharge extinguishing voltage (PDEV) means that when partial discharge occurs, the voltage continues to rise and a severe partial discharge begins to appear, at this time, the voltage value is gradually reduced, when the discharge amount is less than a certain value, the partial discharge phenomenon has just disappeared, record the voltage value at this time PDEV. Figure 4 shows typical pd signals measured during the test. The red line in Fig. 4 is the waveform of the UHF detection discharge signal, the peak of which represents the discharge signal appearing here.

## Experimental results and analysis

### PDIV

**Figure 5:**
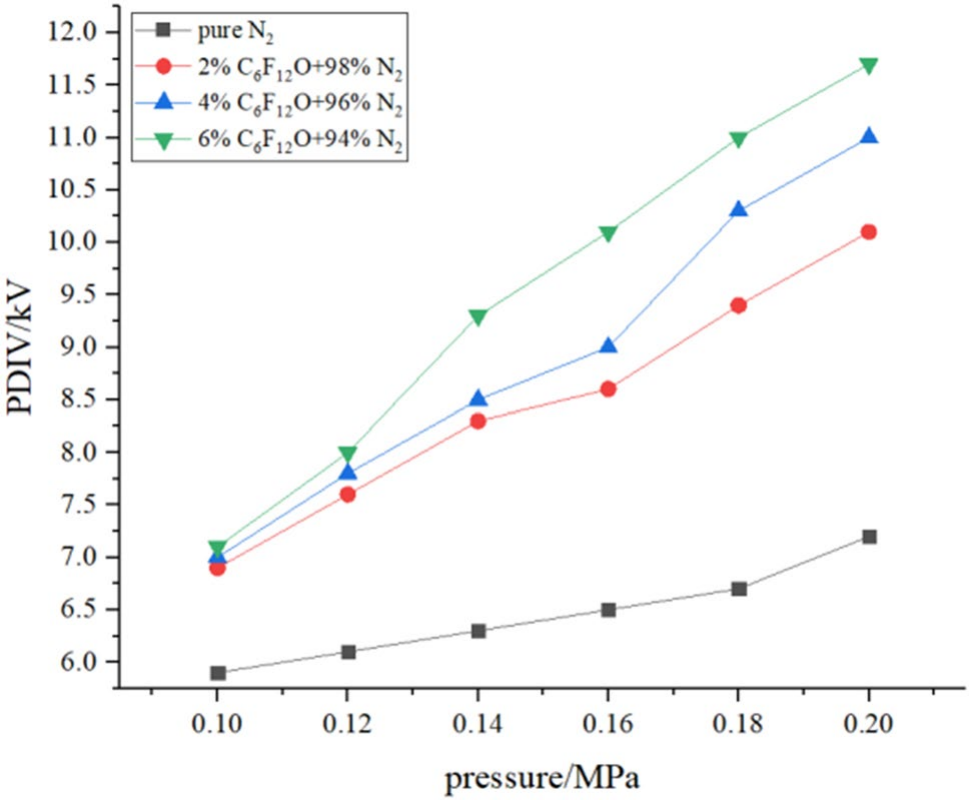
PDIV with the pressure.

**Figure 6:**
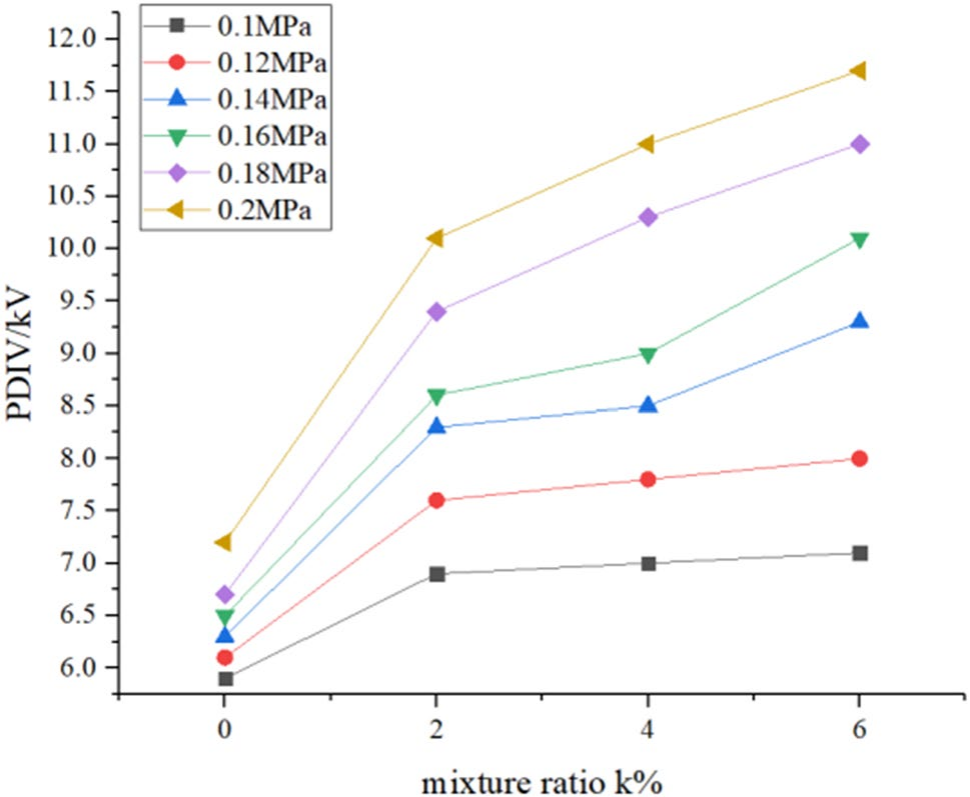
PDIV with the mixing ratio.

The case where the PDIV of the mixed gas changes with the pressure under different mixing ratios and the case where the PDIV of the mixed gas changes with the mixing ratio under different pressures are shown in Figs. 5 and 6, respectively. Each experiment adopts the step-by-step voltage division method, and the voltage is applied slowly at a constant speed from 0 kV. After the voltage is stable, observe the signal of the oscilloscope. When the voltage is high, it will cause the pin-board electrode to break down. At this time, the breakdown voltage is recorded, the voltage regulator is self-protected, the voltage drops to zero, and it is turned off. Then repeat the above operation for 5 tests, and take the average of all test results as the final result under this condition.

It can be seen from the figure that the PDIV value of the gas increases with the increase of the pressure and the gas mixing ratio. The pure N_2_ has a PDIV value of 5.9 kV at 0.1 MPa and 7.2 kV at 0.2 MPa, with an increase of 1.3 kV. The PDIV value of the 2% mixed gas was 6.7 kV at 0.1 MPa and 10.1 kV at 0.2 MPa, with an increase of 3.4 kV. The PDIV values of the mixture of 4% and 6% mixed gas increased by 4 kV and 4.6 kV respectively in this changing pressure. It can be concluded that the mixed gas of the high mixing ratio increases the PDIV value faster as the pressure changes.

The relationship between the starting voltage and the pressure is fitted by the formula

1 $$ U_{PDIV} = AP + B. $$

*U*_*PDIV*_ is the PDIV value, *A* is the slope reflecting the rate of change of PDIV value with pressure, and *P* is the pressure. The PDIV value of the mixed gas exhibits a positive correlation with the pressure. The larger the value of *A*, the more obvious the PDIV value of the mixed gas is affected by the pressure. The results calculated after fitting are: *A*_0%_ = 12.14; *A*_2%_ = 32.43; *A*_4%_ = 40; *A*_6%_ = 46.86. From this, it can be concluded that the higher the mixing ratio, the greater the influence of the pressure on the gas mixture.

In Fig. 6, when 2% C_6_F_12_O is added, the PDIV value of the gas increases rapidly. After adding 4% C_6_F_12_O gas, the gas PDIV value increases slowly. And the higher the pressure, the faster the PDIV value increases. It can be concluded that the PDIV value will increase faster after N_2_ is added with C_6_F_12_O under high pressure, but as the mixing ratio increases the speed becomes slower at a certain pressure.

Table 2 shows the ratio of the PDIV value at different pressures of each group of mixed gases to the PDIV value of N_2_, reflecting the increase in the partial discharge starting voltage of the mixed gas obtained after the addition of C_6_F_12_O. It can be seen from the table that as the pressure and the mixing ratio increase, the ratio of the PDIV value of each mixed gas to the PDIV value of N_2_ is higher. At the same mixing ratio, as the pressure increases, the ratio of the PDIV value of the mixed gas to the PDIV value of N_2_ gradually increases. From Table 2, the ratio of 2% mixed gas to pure N_2_ increases from 1.14 times at 0.1 MPa to 1.40 times at 0.2 MPa with the increase of pressure, at the same time, when the mixing ratio is 6%, the ratio of PDIV of the mixed gas to PDIV of pure N2 increases from 1.20 times of 0.1 MPa to 1.63 times of 0.2 MPa. So the mixed gas of high mixed ratio increases the PDIV value more obviously with the increase of pressure, which consistent with the results of the previous linear. At the same pressure, as the mixed ratio increases, the ratio of the PDIV value of the mixed gas to the PDIV value of N_2_ also gradually increases. When the pressure is 0.1 MPa and the mixing ratio of the mixed gas increases from 2 to 6%, the ratio of the PDIV value of the mixed gas to the PDIV value of N_2_ is from 1.14 to 1.20, an increase of 5.3%; and when the pressure is 0.2 MPa, the data changes from 1.40 to 1.63, an increase of 16.4%. From this it can be concluded that the addition of C_6_F_12_O to N_2_ will result in a more significant increase in the PDIV value of the mixed gas at higher pressure.

**Table 2:** PDIV ratio of C_6_F_12_O mixed gas to pure N_2_.

Presure/MPa	Mixing ratio			
	0%	2%	4%	6%
0.2	1	1.40	1.53	1.63
0.18	1	1.40	1.54	1.64
0.16	1	1.32	1.38	1.55
0.14	1	1.32	1.35	1.48
0.12	1	1.25	1.28	1.31
0.1	1	1.14	1.19	1.20

In summary, when the pressure is higher than 0.16 MPa and the mixing ratio is higher than 2%, the PDIV value of the mixed gas is increased by 50% or more compared to pure N_2_.

### PDEV

**Figure 7:**
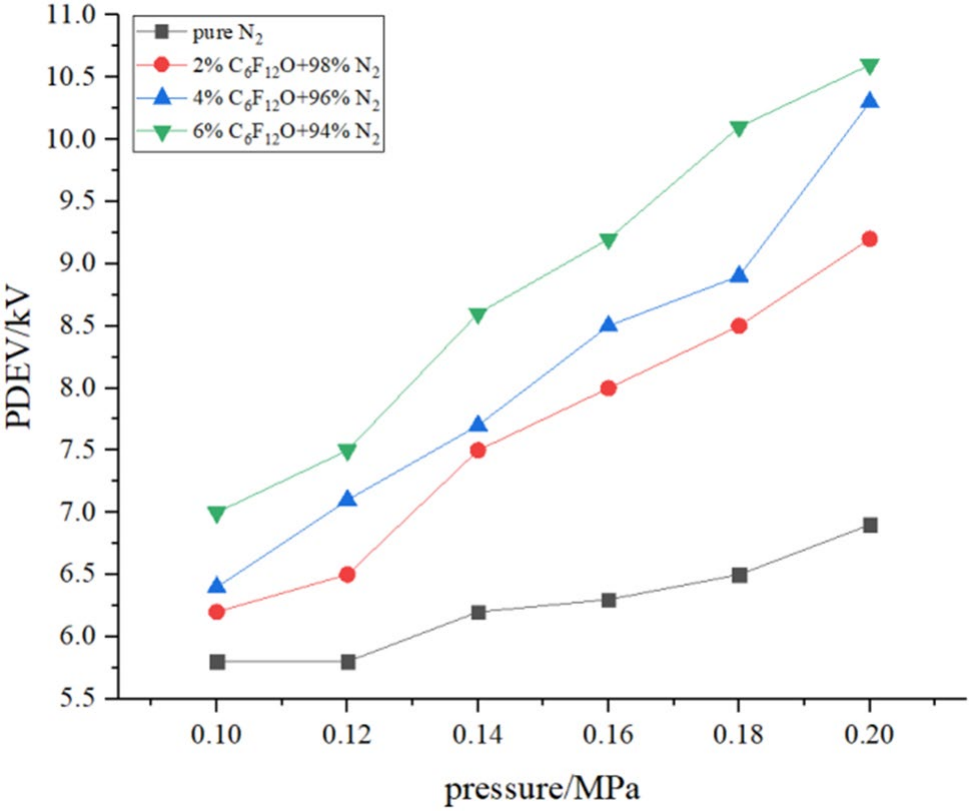
PDEV with the pressure.

**Figure 8:**
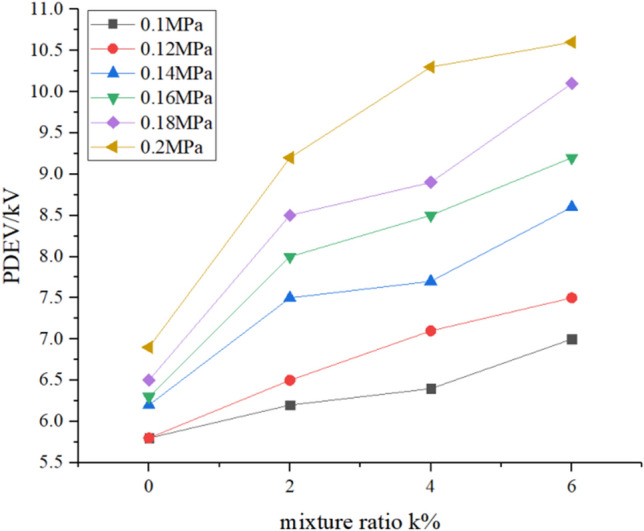
PDEV with the mixing ratio.

In this experiment, the measurement results of the partial discharge extinction voltage according to the experimental procedure are shown in Figs. 7 and 8. The figures show the change of PDEV value with pressure under different mixing ratios and the change of PDEV value with mixing ratio under different pressures.

In Figs. 7 and 8, similar to the change of gas PDIV value, as the mixing ratio and pressure increase, the gas PDEV value gradually increases. For pure N_2_, its PDEV value is 5.8 kV at 0.1 MPa, and its PDEV value is 6.9 kV at 0.2 MPa, an increase of 1.1 kV. For 2% C_6_F_12_O, its PDEV value is 6.2 kV at 0.1 MPa, and its PDEV value is 9.2 kV at 0.2 MPa, an increase of 3 kV. When the mixing ratio is 4%, the PDEV value of the mixed gas at 0.1 MPa is 6.4 kV, which is not significantly improved compared to the 2% mixed gas. When it reaches 0.2 MPa, it increases to 10.3 kV and increases by 3.9 kV. When the mixing ratio reaches 6%, the PDEV value of the mixed gas is 7 kV at 0.1 MPa, which is a relatively large increase compared to the ratio of 2% and 4%. At 0.2 MPa, the PDEV value of the mixed gas is 10.6 kV, which increases 3.6 kV. Therefore, the addition of C_6_F_12_O increases the PDEV value of N_2_, while the sensitivity to pressure increases slightly.

According to the formula (2), linearly fit each curve in Fig. 7 to study the linear relationship between the PDEV value of various mixed gases and the pressure.

2 $$ U_{PDEV} = A^{\prime}P + B^{\prime}, $$

where *U*_*PDEV*_ is the partial discharge quenching discharge voltage value, and *A*′ is the slope. The values of *A*′ calculated for each curve are: *A*′_0%_ = 11, *A*′_2%_ = 30.71, *A*′_4%_ = 36.71, *A*′_6%_ = 37.71. The values of the four curves *A*′ are all positive values, that is, the PDEV voltage value of each group of gases is positively correlated with the pressure. After adding C_6_F_12_O, the influence of pressure on the PDEV value of the mixed gas increases sharply, and the higher the mixing ratio of the mixed gas, the greater the PDEV value is affected by the pressure. The PDEV value of the mixed gas with a mixing ratio of 4% and 6% is approximately the same under the influence of pressure.

From Fig. 8, adding 2% C_6_F_12_O will increase the PDEV value of N_2_, and as the pressure is increased, the magnitude of the increase also increases accordingly. However, the PDEV value of the mixed gas with a mixing ratio of 4% relative to the mixed gas of 2% has not increased much. At 0.14 MPa and 0.18 MPa, the PDEV values of the two mixed gases are almost equal. That is, as the mixing ratio continues to increase, the increasing trend of the PDEV value of the mixed gas gradually slows down.

Table 3 lists the ratio of the PDEV value of each group of mixed gas to the PDEV value of pure N_2_, further showing the partial discharge extinction voltage characteristics of the mixed gas. In the case where the mixing ratio is constant, the ratio of the PDEV value of the mixed gas to the PDEV value of N_2_ gradually increases as the pressure increases. When the mixing ratio is 2% and the pressure is from 0.1 to 0.2 MPa, the ratio of the PDEV value of the mixed gas to the PDEV value of N_2_ increases from 1.07 to 1.33; At a mixing ratio of 6%, this value increased from 1.21 to 1.54. So the higher the mixing ratio, the higher the ratio of the PDEV value of the mixed gas to the PDEV value of N_2_. At a fixed pressure, the increase of the mixing ratio for the increase of the N_2_^’^s PDEV value is as follows. At 0.1 MPa, the ratio of the PDEV value of the mixed gas to the PDEV value of N_2_ changes from 1.07 to 1.21 as the mixing ratio increases. At 0.2 MPa, the ratio of the PDEV value of the mixed gas to the PDEV value of N_2_ increases from 1.33 to 1.54 as the mixing ratio increases. It can be concluded that the higher the pressure, the higher the ratio of the PDEV value of the mixed gas to the PDEV value of pure N_2_, and the greater the increase in the PDEV value of the mixed gas relative to the pure N_2_. The PDEV value of 6% of the mixed gas at 0.18 MPa and above can reach more than 1.5 times of N_2_ under the same conditions.

**Table 3:** PDEV ratio of C_6_F_12_O mixed gas to pure N_2_.

Pressure/MPa	Mixing ratio			
	0%	2%	4%	6%
0.2	1	1.33	1.49	1.54
0.18	1	1.31	1.37	1.55
0.16	1	1.27	1.35	1.46
0.14	1	1.21	1.24	1.39
0.12	1	1.12	1.22	1.29
0.1	1	1.07	1.10	1.21

### Comparison of partial discharge voltage and breakdown voltage of mixed gas

This section compares the partial discharge characteristics and breakdown characteristics of the gas. Tables 4 and 5 respectively list the ratio of partial discharge inception voltage and partial discharge extinction voltage to breakdown voltage of the mixed gas under different mixing ratios and different pressure conditions.

**Table 4:** PDIV value/breakdown voltage value.

Pressure/MPa	Mixing ratio			
	0%	2%	4%	6%
0.2	0.62	0.37	0.34	0.36
0.18	0.58	0.37	0.34	0.35
0.16	0.57	0.38	0.32	0.34
0.14	0.58	0.40	0.33	0.35
0.12	0.61	0.41	0.34	0.34
0.1	0.69	0.40	0.35	0.34
Average value	0.61	0.39	0.34	0.35

**Table 5:** PDEV value/breakdown voltage value.

Pressure/MPa	Mixing ratio			
	0%	2%	4%	6%
0.2	0.59	0.34	0.32	0.32
0.18	0.57	0.33	0.30	0.33
0.16	0.55	0.35	0.31	0.31
0.14	0.57	0.36	0.30	0.33
0.12	0.58	0.35	0.31	0.32
0.1	0.67	0.37	0.32	0.33
Average value	0.59	0.35	0.31	0.32

Table 4 shows that the ratio of the pure N_2_’s PDIV value to the breakdown voltage value is concentrated around 0.6 at 0.1–0.2 MPa, when the mixing ratio is 2%, the ratio of the mixed gas’s PDIV value to the breakdown voltage is about 0.4 at 0.1–0.2 MPa, so the partial discharge voltage of the mixed gas is already smaller than the breakdown voltage value under the same conditions. When the mixing ratio is 4% and 6%, the ratio of the mixed gas’s PDIV value to the breakdown voltage value is about 0.34 at 0.1–0.2 MPa. It shows that under this mixing ratio condition, the partial discharge starting voltage is great smaller than the breakdown voltage. It can be concluded that when the pure N_2_ is partially discharged, the voltage value may be relatively close to the breakdown value. After adding C_6_F_12_O, the difference between the partial discharge voltage and the breakdown voltage of the gas increases significantly.

It can be seen from Table 5 that the ratio of the pure N_2_’s PDEV value to the corresponding breakdown voltage value is 0.59 at 0.1–0.2 MPa, which is relatively close to the above-mentioned PDIV value. When the mixing ratio is 2%, the ratio of the mixed gas’s PDEV value to the breakdown voltage value under the corresponding conditions is about 0.35 at 0.1–0.2 MPa. When the mixing ratio is 4% and 6%, this value is 0.31 and 0.32 respectively. The addition of C_6_F_12_O mixed gas can reduce the ratio of the pure N_2_’s partial discharge extinction voltage to the breakdown voltage under corresponding conditions.

## Application analysis of C_6_F_12_O mixed gas insulation equipment

The smaller the partial discharge of the insulating equipment, the better the performance. The current standard stipulates that the level of partial discharge is mainly considering the usage time under the current common process conditions and under normal operating conditions. Through the experimental data analysis of the partial discharge characteristics of the C_6_F_12_O mixed gas in this paper, the results show that the discharge amount of the C_6_F_12_O mixed gas is small. However, the long-term partial discharge will cause destructive effects on the insulating material, and eventually lead to insulation equipment failure. Therefore, for new equipment, the discharge capacity should not exceed the specified value. When the discharge amount exceeds l times of the standard, although the impact on the equipment is very small, it cannot be ignored. When the discharge amount exceeds 1–4 times of the standard, it is necessary to analyze the possible causes and monitor the operation. If it exceeds 10 times the standard value or even greater, it means that there may be serious hidden faults in the insulation equipment. Faults are usually exposed between 2 months and 2 years, and various invisible faults are often unable to be detected by other insulation tests. Measuring the partial discharge experimental data of C_6_F_12_O mixed gas can provide data for monitoring equipment, which can be found and solved in the early stage of partial discharge of electrical equipment, so as to prevent equipment failure caused by long-term partial discharge of electrical equipment. At the same time, the best gas mixing ratio and pressure data can also be determined, which lays a theoretical foundation for practical engineering application. Therefore, the partial discharge experiment of C_6_F_12_O mixed gas can provide technical reference for preventing electrical equipment faults. And it can ensure the safe operation of the equipment, which has a certain engineering reference significance^12^.

## Conclusion

1. The partial discharge inception voltage and partial discharge extinction voltage of the C_6_F_12_O mixed gas gradually increases with the increase of the mixed gas and the mixing ratio. The partial discharge characteristic of mixed gas grows slowly with the increase of mixing ratio, and the pressure has a greater influence on the gas with a higher mixing ratio.
2. The breakdown voltage of pure N_2_ is greatly improved after adding C_6_F_12_O, when the mixing ratio is 4% at a pressure of 0.18 MPa or the mixing ratio is 6% at a pressure of 0.16 MPa, the breakdown voltage can be achieved 2.5 times or more than the pure N_2_.
3. When comparing the partial discharge voltage with the breakdown voltage value, it was found that the partial discharge voltage value and the breakdown voltage value of the mixed gas after the addition of C_6_F_12_O were smaller than that of pure N_2_.
4. The partial discharge of the C_6_F_12_O mixed gas phase is small, which can provide technical reference for the safe operation of the C_6_F_12_O mixed gas equipment and the prevention of electrical equipment failures, it has engineering significance.
